# Impact of *Viburnum opulus* L. Fruit Extracts on the Physicochemical, Sensory, and Bioactive Properties of Wheat Waffles

**DOI:** 10.3390/molecules30244677

**Published:** 2025-12-05

**Authors:** Begümhan Ömeroğlu Gülada, Urszula Złotek, Urszula Gawlik, Dariusz Kowalczyk, Duygu Taşkın, Turgut Taşkın, Anna Jakubczyk, Fatma Esra Güneş

**Affiliations:** 1 Department of Nutrition and Dietetics, Faculty of Health Sciences, Balikesir University, Çağış Yerleşkesi, Bigadiç, 10463 Balıkesir, Turkey; begumhan.gulada@balikesir.edu.tr; 2 Department of Biochemistry and Food Chemistry, Faculty of Food Sciences and Biotechnology, University of Life Sciences in Lublin, Skromna 8, 20-704 Lublin, Poland; urszula.gawlik@up.edu.pl (U.G.); dariusz.kowalczyk@up.edu.pl (D.K.); anna.jakubczyk@up.edu.pl (A.J.); 3 Department of Analytical Chemistry, Faculty of Pharmacy, University of Health Sciences, Tibbiye Cad. No. 38, Üsküdar, 34668 Istanbul, Turkey; duygu.taskin@sbu.edu.tr; 4 Department of Pharmacognosy, Faculty of Pharmacy, Marmara University, Başıbüyük Yolu, Başıbüyük, 34854 Istanbul, Turkey; turguttaskin@marmara.edu.tr; 5 Department of Nutrition and Dietetics, Faculty of Health Sciences, Medeniyet University, Şehit Hakan Kurban Cd. No. 44, Kartal, 34862 Istanbul, Turkey; fatmaesra.gunes@medeniyet.edu.tr

**Keywords:** *Viburnum opulus* fruit, European cranberrybush, polyphenols, waffles, enriched food, functional food

## Abstract

This study aimed to compare the phenolic profiles and bioactive properties of powdered aqueous (AVOE) and methanolic (MVOE) *Viburnum opulus* L. fruit extracts and compare the effect of increasing concentrations of these extracts on the physicochemical, sensory, and bioactive properties of wheat waffles. The polyphenol content and bioactivity of the extracts and waffles were assessed based on their water–ethanol and phosphate-buffered saline extracts, as well as on samples after in vitro digestion. MVOE and MVOE-enriched waffles showed superior, statistically significant antioxidant and anti-inflammatory activity compared to AVOE and AVOE-enriched waffles. Functional analyses revealed that the enrichment level affected water absorption, texture, and sensory perception, with reduced acceptability at high aqueous extract levels. Overall, methanolic extract enrichment provided the best balance between functionality and bioactivity, and waffles enriched with the highest amount of MVOE were characterized by having the highest potentially bioavailable polyphenol fraction (17.47 ± 1.01 mg GAE/gDW) as well as the strongest ability to inhibit LOX and XO activity (EC_50_ = 8.55 ± 0.16 and 9.25 ± 0.01 mgDW/mL, respectively) with simultaneous high consumer acceptability. These results confirm the applicability of VO extracts in food formulations and potential health benefits, offering a basis for future clinical studies on VO-enriched functional foods.

## 1. Introduction

*Viburnum opulus* L. (VO) is a deciduous shrub, 2–4 m in height, belonging to the *Caprifoliaceae* family [1]. It grows naturally across Europe, North Asia, North Africa, and Russia [2]. The fruit is known as “Gilaburu” or “Gilabou” in Turkish; “Guelder Rose”, “Snowball Bush”, “European Cranberry”, “Highbush Cranberry”, “American Cranberrybush”, “Stagbush”, and “Cranberry Viburnum” in English; “Gemeiner Schneeball” in German; and “Kalina Koralowa” in Polish [3]. VO fruit contains abundant flavonoids and phenolic acids, including chlorogenic, caffeic, *p*-coumaric, gallic, protocatechuic, syringic, and ellagic acids [4], which confer high antioxidant capacity [5,6,7,8]. Traditionally, VO fruit or its juice has been used in Turkish folk medicine for conditions such as renal calculi, hypertension, cramps, and menstrual pain. Moreover, scientific studies have reported beneficial effects against colon cancer and testicular damage, and demonstrated antiurolithiatic, antinociceptive, antiproliferative, antiallergic, antimicrobial, antiviral, and anti-inflammatory activities [6,9,10,11,12,13,14,15,16,17,18,19]. However, the fruit and its juice possess an unpleasant odor caused by valeric acid, which limits consumer acceptance [20].

Antioxidants play a crucial role in human health by reducing, eliminating, or mitigating the effects of oxidative reactions involved in diseases associated with oxidative stress, such as cancer, neurodegenerative disorders, and inflammatory bowel disease [21]. Efficient antioxidant defense mechanisms are essential for maintaining vital cellular functions [22]. Polyphenols, a diverse group of bioactive compounds including flavonoids and non-flavonoids, are abundant in plant-based foods and exert antioxidant, anti-inflammatory, anticarcinogenic, and immunomodulatory effects [23,24]. Rich dietary sources include fruits, vegetables, nuts, olives, and food products such as juice, jam, or wine, which contribute to the prevention of chronic diseases [25]. Epidemiological and clinical studies suggest that diet can play a crucial role in preventing chronic and inflammatory diseases, as well as modifying their course [26,27,28,29]. Recently, many people around the world have shown interest in enriched food products to improve the quality of their diets and access high-quality nutrients. Lately, many experimental and clinical studies have been conducted evaluating food products enriched with fruits, vegetables, or herbs with high antioxidant capacity [30,31,32,33]. Despite the global increase in processed food consumption, there is growing consumer demand for high-quality and functional foods. Manufacturers have responded by developing innovative products such as cookies, bars, breads, and soup mixes enriched with bioactive ingredients. These functional foods are designed to provide desirable and healthier alternatives to conventional products [34,35]. Baked goods, in particular, are especially suitable for enrichment because of their popularity and frequent consumption. Accordingly, numerous studies have explored the incorporation of antioxidant-rich fruits and plants into food formulations. Recent research has not only examined the functional properties of such enriched products but has also assessed the structural characteristics that influence consumer preference [30,34]. Furthermore, clinical intervention studies have increasingly investigated the health effects of antioxidant-enriched foods in both patient and healthy populations [30,31,36,37,38,39].

While previous reviews have provided a general overview of antioxidant-enriched bakery products, recent research has specifically examined the incorporation of fruit-based polyphenols in baked goods like cookies, bread, and waffles. These studies have evaluated the thermal stability and sensory impact of polyphenols in bakery matrices [40,41]. Fruit pomace or concentrated fruit extracts, such as apple, sour cherry, or berry by-products, have been shown to enhance antioxidant capacity without significantly compromising sensory acceptability [42,43]. Furthermore, improved baked goods have demonstrated increased phenolic content, antioxidant activity, and even enzyme inhibition (e.g., α-amylase and α-glucosidase), highlighting both functional and nutritional benefits [44].

Despite its exceptionally high antioxidant and anti-inflammatory potential, the unpleasant odor of VO fruit limits its consumer acceptance, which may explain why it has been underutilized in food improvement. Furthermore, to date, no studies have investigated baked goods enriched specifically with extracts from VO fruit. Therefore, this study was designed to fill this gap by evaluating the effects of VO fruit extracts on the physicochemical, sensory, and bioactive properties of wheat waffles. The research hypothesis is that the addition of VO extracts improves the bioactive properties of waffles. This study aimed to (i) compare the phenolic profiles and bioactive properties (antioxidant and anti-inflammatory activities) of powdered aqueous (AVOE) and methanolic (MVOE) VO fruit extracts and then (ii) compare the effect of increasing concentrations of these extracts on the physicochemical, sensory, and bioactive properties (antioxidant and anti-inflammatory activities) of wheat waffles. The polyphenol content and bioactivity of the powdered extracts and waffles were assessed based on their water–ethanol (as control chemical extracts) and phosphate-buffered saline (PBS) extracts (as control extracts for samples after simulated digestion, treated as samples before in vitro digestion), as well as on samples obtained after in vitro digestion.

## 2. Results

### 2.1. Qualitative Analysis of Phenolic Compounds in AVOE and MVOE

The phenolic compounds identified by LC-ESI-tandem MS in the extracts are summarized in Table 1 and Table 2, respectively, with the corresponding chromatograms presented in Supplementary Figures S1–S7. By comparing molecular weights and their fragments with authentic standards and data from the literature, malic acid (non-phenolic acid), quinic acid, chlorogenic acid, and catechin were identified in both extracts. Malic acid was characterized by a molecular ion at *m*/*z* 133 and a fragment ion at *m*/*z* 115. Quinic acid was confirmed by the presence of fragment ions at *m*/*z* 127 [M–H–H_2_O–H_2_O–CO]^−^ and a deprotonated molecular ion at *m*/*z* 191. The compound with an [M–H]^−^ ion at *m*/*z* 354 and a fragment ion at *m*/*z* 191 in the MS/MS spectra corresponded to chlorogenic acid. Catechin, with a molar mass of 290, was identified in a negative mode, yielding a [M–H]^−^ ion at *m*/*z* 289 and a characteristic fragment peak at *m*/*z* 245 (Table 1 and Table 2). In addition, cinchonain was tentatively identified in both extracts based on reports from the literature [45], while tricin and procyanidin derivatives were also tentatively identified in the AVOE (Table 1), supported by previous studies [46,47].

### 2.2. Physicochemical Properties of Waffles

**Figure 1:**
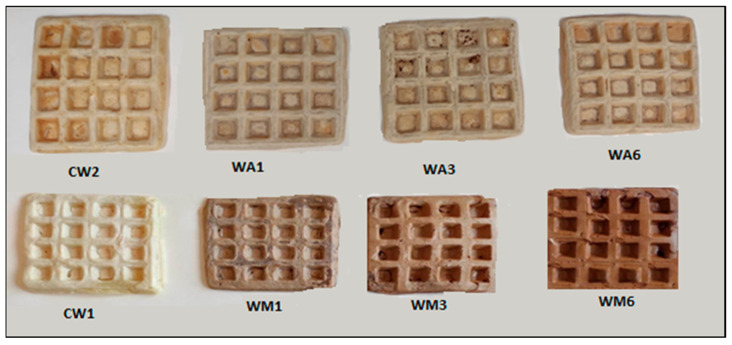
The general appearance of control waffle (CW1), control waffle with maltodextrin (CW2), and waffles with increasing content of powdered aqueous (WA1, WA3, WA6) and methanolic (WM1, WM3, WM6) *Viburnum opulus* fruit extracts.

Figure 1 presents the general appearance of the obtained waffles. All waffles maintained the characteristic square grid structure typical of this product. The substitution of water with maltodextrin (CW2) resulted in a slightly darker surface color compared to the general control (CW1). As expected, waffles enriched with the extracts were darker than the controls. A gradual darkening of the waffles’ surface with increasing MVOE content (WM1, WM3, WM6) was observed, whereas in the case of waffles with varying AVOE concentrations (WA1, WA3, WA6), the color was visually quite comparable. The WM series was noticeably darker in comparison to the WA (Figure 1).

The results of instrumental color analysis are presented in Table 3. Replacing water with maltodextrin (CW2) had no significant effect on lightness (L*), but increased the redness (a*) and yellowness (b*) compared to the control (CW1). No significant differences in L* values were observed between the control waffles and AVOE-enriched waffles, regardless of concentration (L* = 65.34–69.01). In line with the visual observations, the MVOE-enriched waffles showed an increase in darkness, redness, and yellowness in a dose-dependent manner. Regardless of the concentration, these waffles were darker (L* = 29.19–36.85) than the other samples, and at medium and high extract incorporation levels, they were also the most red (*p* < 0.05). Because the WA series was not as red as the WM series, they appeared more yellow (Table 3).

While the cutting force test revealed no significant difference between samples with and without maltodextrin, the penetration test demonstrated that the addition of maltodextrin nearly doubled the hardness of the waffles (Table 3). Based on PS values, the addition of extracts, regardless of concentration, did not cause textural changes in the waffles compared to CW1. Meanwhile, the cutting test revealed that low and medium levels of addition led to a slight softening of the waffle structure (which could be unfavorable for sensory attributes), whereas at the highest concentration this effect was no longer observed.

The WAC of the waffles ranged from approximately 322 to 396 g/100 g DW (Table 3). Only waffles enriched with the lowest levels of the extracts (WA1 and WM1) exhibited significantly higher water absorption capacity compared to the other samples. The OAC of all waffle samples was relatively consistent, ranging from 226.16 to 259.31 g/g DW (Table 3), with no significant differences between plain control, maltodextrin-containing, or extract-enriched waffles.

### 2.3. Sensory Attributes of Waffles

The sensory evaluation of the waffles revealed that neither the type nor the level of extract enrichment influenced their acceptability (*p* < 0.05) (Table 4). This was due to relatively large differences in the perception of the waffles among the panelists, which consequently resulted in high standard deviations. Nevertheless, it can be seen that the general control (CW1) exhibited moderate scores across all attributes (4.33–5.83), while the maltodextrin-containing control (CW2) showed slightly higher ratings for taste and aroma (6.50), indicating a modest improvement in sensory quality. Among the WA series, acceptability decreased with increasing extract levels; WA6, containing the highest AVOE concentration, received the lowest scores for taste and overall impression (3.58 and 4.08, respectively). As for the MVOE-enriched waffles, WM3 and WM6 tended to exhibit slightly higher scores for taste and overall impression compared to the control, indicating generally good acceptability. Overall, moderate levels of extract enrichment, particularly MVOE, tended to maintain or improve sensory acceptability, whereas higher AVOE levels were associated with reduced consumer appeal.

### 2.4. Content of Phenolic Compounds in VO Extracts and Waffles

In the ethanolic extracts, the TPC of all waffle samples except WA1 showed statistically different contents (*p* < 0.05) compared to control waffle types. Total phenolic content in the ethanolic extracts of waffles increased proportionally with the increase in the amount of AVOE and MVOE additions (Table 5). The highest TPC amount among all waffles was determined to be in WM6 (7.58 ± 0.68 mg GAE/gDW). It was determined that the TPCs of both fruit extracts were statistically similar. While the TFC did not change in waffles containing AVOE (*p* < 0.05), it was statistically different in all waffle types containing MVOE (*p* < 0.05). When waffles containing two different types of extracts with the same content were compared, the TFC of WM6 (14.50 mgQE/gDW) was determined to be 3.85 times higher than the TFC of WA6 (3.76 mgQE/gDW). There was a difference in the TFC between the extract types (*p* < 0.05). TFC was found to be higher in MVOE than in AVOE. PAC did not change with the addition of either type of extract compared to control waffles (*p* < 0.05). However, there was a difference in PAC between the extract types (*p* < 0.05). PAC was observed to be higher in AVOE than in MVOE (142.31 μg CAE/gDW vs. 30.60 μg CAE/gDW, respectively).

In the PBS extracts, the TPC among the waffle samples ranged from 0.81 mg GAE/gDW (CW1) to 5.78 mg GAE/gDW (WM6). The content of these compounds increased in direct proportion to the VOFE (*Viburnum opulus* L. fruit extract) concentration in the waffles (Table 5). TFC in PBS extracts was found to be higher in AVOE than in MVOE (42.19 mgQE/gDW vs. 7.98 mgQE/gDW, respectively). PAC increased compared to control waffles with the addition of both 6 g VOFEs (*p* < 0.05). Furthermore, statistically significant differences in PAC were observed between WME3 and WME6 (*p* < 0.05). PAC was observed to be higher in AVOE than in MVOE (69.90 μg CAE/gDW vs. 42.17 μg CAE/gDW, respectively).

After in vitro gastrointestinal digestion simulation (GID), TPC was significantly higher in MVOE and MVOE-added waffles compared to ethanol and PBS extracts, particularly in MVOE (Table 5). Flavonoid content after digestion also increased with increasing amounts of both VOFEs in waffles. A statistically significant increase was observed in all waffle samples compared to the control. The highest TFC was determined in MVOE (464.15 mgQE/gDW). The highest PAC was determined in MVOE (231.56 μg CAE/gDW). These compounds were determined as 44.48 μg CAE/gDW in AVOE. It should be noted that in the case of samples after in vitro digestion, PAC in MVOE was 5.49 times higher than in AVOE (Table 5).

### 2.5. The Antioxidative Activities of VO Extracts and Waffles

The radical scavenging activities (ABTS, DPPH, OH), reducing power (FRAP), and metal chelating capacity (CHP) of the waffle samples and the two different types of fruit extracts which were used in waffle production are shown in Table 6.

ABTS radical scavenging activity of ethanolic extracts from the studied waffles increased with increasing amounts of enrichment (*p* < 0.05). Enrichment did not change ABTS radical scavenging activity in the case of the PBS and GID extracts (*p* < 0.05). In the case of the ethanolic and PBS extracts, ABTS radical scavenging activity was the highest in the AVOE sample (88.40 mgTE/gDW and 982.75 mgTE/gDW, respectively), while in the case of GID extracts, it was found in MVOE (5266.10 mgTE/gDW) (*p* < 0.05). DPPH radical scavenging activity did not change with amount of enrichment for both extract types, except for ethanolic and PBS extracts from the WM6 sample. However, enrichment with AVOE did not change DPPH scavenging activity in the case of GID samples. As the amount of enrichment with MVOE increased, so did DPPH radical scavenging activity (*p* < 0.05). There were no differences in DPPH scavenging activity between AVOE and MVOE in PBS extracts (*p* < 0.05). OH radical scavenging activity was negatively affected by the addition of both types of fruit extracts and decreased compared to the control (ethanolic extracts) (*p* < 0.05). In PBS extracts, enrichment did not alter OH radical scavenging activity, and no difference was found between the VO extracts (*p* < 0.05). In the case of samples after in vitro digestion, OH radical scavenging activity increased compared to the control with the addition of both types of fruit extracts (*p* < 0.05). However, the MVOE extract had the highest OH radical scavenging activity (436.92 TE mg/gDW).

Ferric reducing antioxidant power (FRAP) increased in WA3 and WA6 samples in comparison to the control in the case of ethanolic extract, while MVOE addition caused an increase in this activity in all cases of extracts (*p* < 0.05). When the FRAP of the two fruit extracts (AVOE and MVOE) was compared (ethanolic extracts and samples after digestion), MVOE had the highest activity (211.63 mgTE/gDW and 318.18 mgTE/gDW, respectively).

In the case of PBS extracts, FRAP did not change in samples with the addition of AVOE (*p* < 0.05), while it increased in samples from waffles with the addition of MVOE compared to the control (*p* < 0.05). However, the amount of MVOE added did not change FRAP activity (*p* < 0.05). Both fruit extracts (AVOE and MVOE) were found to have similar FRAP activity. In the case of samples after in vitro digestion, FRAP did not change in samples with the addition of AVOE (*p* < 0.05), but it increased in samples with the addition of MVOE (*p* < 0.05). Among the two fruit extracts, MVOE had the highest FRAP activity (318.18 mg TE/gDW). Iron chelation (CHP) increased in samples with the addition of AVOE compared to the control (ethanolic extracts), but there was no difference between the amounts of addition. This activity increased in samples with the addition of MVOE compared to the control, and the WM6 sample was found to be higher than the WM1 and WM3 samples (*p* < 0.05). CHP of both fruit extracts was similar (*p* < 0.05). In case of PBS extracts, improvement by the addition of both types of fruit extracts increased CHP (*p* < 0.05). Among all the enriched waffles, WM6 had the highest CHP (6963.10 mg EDTA/gDW). In PBS extracts, the two different fruit extracts differed in CHP, with MVOE exceeding AVOE (6817.6 mg EDTA/gDW and 6302.08 mg EDTA/gDW, respectively). In the case of samples after in vitro digestion, enrichment with both extracts (AVOE and MVOE) increased CHP, but it increased as the amount of AVOE increased, while the amount of MVOE addition did not change CHP. It should be added that in samples after digestion, the highest CHP activity was detected in MVOE (9403.60 mgEDTA/gDW).

### 2.6. Potential Anti-Inflammatory Activities of VO Extracts and Waffles

LOX-inhibitory activity increased slightly with increasing addition of both studied extracts in all kinds of extracts (ethanolic, PBS, and GID) (Table 7). The lowest LOX-inhibitory activity was determined for ethanolic extracts, while the highest values of this activity were calculated in MVOE and its waffles after in vitro digestion. The highest XO inhibitory potential was found in MVOE and its waffles for GID extracts, while the lowest inhibition was determined in PBS extract from the WA1 sample (the lower the IC_50_ value, the stronger the inhibition of XO activity).

### 2.7. Ranking of Antioxidant Capacity Following In Vitro Digestion

The developed products and extracts were ranked from one to ten based on their phenolic content, radical scavenging activity, iron-reducing, and iron-chelating power after in vitro gastrointestinal digestion, and a mean rank and overall performance score were generated accordingly. In this context, the extract with the best antioxidant activity was found to be methanolic VO fruit extract, while the product with the best antioxidant activity was the waffle (WM6) containing 6 g of methanolic VO fruit extract. Within each extract type, the antioxidant ranking of the waffles declined with an increasing extract concentration. Conversely, the control waffles exhibited the lowest antioxidant activity, which placed them at the highest rank in the scoring system. The ranking of antioxidant properties of the products and VO fruit extracts for these different analyses and overall performance is detailed in Table 8.

## 3. Discussion

### 3.1. LC-ESI MS Tandem Analysis of AVOE and MVOE

LC-ESI MS tandem analysis was performed on the fruit extracts (AVOE and MVOE) used in the study before waffle production. Quinine was identified in both extracts using data from the literature [48]. Tricin and procyanidin compounds were also identified in AVOE, respectively, using data from the literature [46,47]. According to the analysis results, malic acid, quinic acid, chlorogenic acid, and catechin were determined in both fruit extracts (AVOE and MVOE). Procyanidin b2 and tricin were detected only in the AVO extract. The type of solvent used during extraction affects the release of different compounds found in the fruit [49]. Indeed, solvent polarity is a key determinant of the qualitative and quantitative phenolic profile: alcohol-based solvents (e.g., methanol) typically extract a broader range and higher amounts of low-to-moderately polar phenolics (flavonoids, chlorogenic and caffeic acids), whereas aqueous extractions favor more polar conjugated or sugar-bound phenolics; therefore, the observed higher TPC/TFC in MVOE is consistent with previous solvent-comparison studies [50,51].

### 3.2. Physicochemical and Sensory Properties of Waffles

Maltodextrin was included only in the CW2 control formulation (as stated in Methods); none of the extract-enriched waffles (WA1, WA3, WA6, WM1, WM3, WM6) contained added maltodextrin. The slightly darker surface of maltodextrin-containing waffles compared to CW1 (Figure 1, Table 3) may be attributed to more intensive Maillard reactions or caramelization during baking. It is possible that maltodextrin underwent thermal degradation into shorter sugars (glucose, maltose, and oligoglucans), thereby promoting browning reactions. The color changes in waffles following the incorporation of fruits are a common and unavoidable effect. The darker color of the extract-enriched waffles resulted from red pigments in VO fruits that were carried over into the extracts [52,53]. The observed dose-dependent darkening and increased redness in WM samples (Table 3) suggest higher pigment content in MVOE compared to AVOE. These findings indicate that both the type and level of extract enrichment influence the color characteristics of waffles, with MVOE having a more pronounced effect than AVOE. Maltodextrin was included in CW2 to standardize solid content and improve handling and reproducibility during batter preparation (it serves as an inert bulking/carrier agent and can act as a fat mimetic or matrix modifier in baked systems), which may also influence browning via increased reducing sugar availability during baking. This functional role of maltodextrin in bakery matrices and its potential to affect texture, water retention, and color has been previously reported [54,55]. Therefore, it was advisable to use maltodextrin as an additional control sample in this work. Furthermore, maltodextrin is commonly used as a carrier to protect and stabilize bioactive extracts during processing and storage, which may be relevant when incorporating concentrated fruit extracts into doughs [56].

To gain a better understanding of the texture of waffles, which have a characteristic shape, two different mechanical tests (cutting vs. penetration) were employed. The observed discrepancies in results (for certain types of waffles) obtained by these tests (Table 3) can be attributed to the different aspects of texture assessed by each method. The penetration test measured localized resistance to force, reflecting the internal structure and rigidity of the waffle, whereas the cutting test evaluated the overall fracture behavior and structural integrity. It was found that maltodextrin strongly reinforced internal rigidity (seen in the PS test) but had a smaller effect on the global cutting resistance. Similarly, the minor softening observed in the cutting test at low and medium extract levels may reflect structural heterogeneities, i.e., disruption of the gluten-starch network leading to slight softening, which was not detected by the puncture test. At high extract levels, this softening effect was likely compensated for by the increased solid content in the product. The findings presented here are consistent with the results of Ekramian et al. [57], who reported that the addition of kiwifruit and fig extracts, as well as fungal protease, significantly affected the texture profile of waffles, particularly by decreasing hardness values depending on the extract concentration.

WAC and OAC are important functional properties that influence waffle texture, mouthfeel, and the ability to retain moisture or fats during consumption, particularly when served with toppings such as fruits, syrup/sauce, ice cream, or whipped cream. Low extract levels (WA1 and WM1) increased WAC, suggesting increased soaking and/or hydrophilicity, which may be undesirable as it could lead to a softer texture and reduced crispness of the waffles. The extracts did not affect fat binding, which could be beneficial for maintaining the waffles’ texture and mouthfeel when served with toppings such as cream, Greek yogurt, or chocolate fondue.

It has been reported in the literature that phenolic compounds found in many foods can cause bitter and astringent tastes [58]. This effect has also been reported in cakes containing VO fruit puree [52]. However, in our study, sensory trends indicate that the enriched waffles were generally well accepted, likely because the extracts contained relatively few off-flavor compounds as compared to the fruit. In future studies, testing waffles supplemented with fruit additions could help to further clarify these differences. Overall, the taste of MVOE-enriched waffles tended to be better accepted than that of AVOE-added. So, AVOE could contain more undesirable compounds, slightly decreasing acceptability. The interaction between individual components of extracts and waffle matrix may also modulate flavor perception, e.g., masking residual bitterness. Our results suggest that, while differences in sensory attributes of the waffles were generally not statistically significant, the type of extract appeared to influence consumer perception.

### 3.3. Phenolic Compounds Content

Recent studies have focused on phenolic compounds and enriched foods containing these compounds to prevent diseases caused by oxidative stress or to modify the course of chronic diseases [59,60,61]. Heat treatment applied during the processing of enriched foods can increase antioxidant capacity by releasing conjugated phenolics. However, since phenolic compounds are sensitive to heat, light, and oxygen, thermal damage can also occur during the processing [62]. Therefore, the antioxidant capacity of foods enriched with plants, fruits, and vegetables is evaluated after the preparation and cooking stages [63,64,65]. The TPC, TFC, and PAC of foods enriched with different types and amounts of fruit vary depending on the solvent used (e.g., ethanol or PBS buffer) and the in vitro digestion process [66,67,68,69].

In this study, changes in the phenolic content of enriched waffles produced using two different types of VO fruit extracts (MVOE and AVOE) were evaluated in ethanol and PBS extracts, as well as in samples after in vitro gastrointestinal digestion. While the TPC value did not change among the fruit extracts following ethanol extraction, the highest TPC was found in MVOE after buffer extraction and after in vitro digestion. In all analyses, the highest TPC was determined in the WM6 sample. Among fruit extracts, TFC was highest in MVOE samples (ethanolic extract and the sample after in vitro digestion) and in the AVOE sample (PBS extract). The highest TFC value in enriched waffles was found in the WM6 sample (ethanolic and PBS extracts), but the amount of enrichment did not change the TFC value in samples after in vitro digestion. PAC was highest in AVOE in ethanolic and PBS extracts, while in samples after digestion, the highest PAC was found in the MVOE sample. While the amount of enrichment did not change the PAC in ethanolic samples from waffles, in the case of PBS extracts, the highest value was found in the WA6 sample, while regarding samples after in vitro digestion, the highest value among enriched waffles was found in the WM6 sample. Similarly to our study, the literature has reported that different solvents and buffers alter the release of TPC, TFC, and PAC [62].

The generally higher TPC, TFC, and PAC values in MVOE and the WM6 group prepared using MVOE may be due to the fruit extract obtained through methanolic maceration being richer in phenolic compounds. While the presence of qualitative compounds depends on the plant part used, the quantitative composition is influenced by the solvent type. A previous study evaluating different types of VO fruit extracts reported that extracts obtained with alcohol-based solvents (ethanol was used in this study) had higher phenolic compound content and antioxidant capacity compared to those treated with water [70]. The overall increase in bioactive compounds and antioxidant capacity after in vitro digestion may be due to the increased bioavailability of other compounds, such as peptides, released during digestion. It has also been reported that phenolic compounds can exhibit greater activity upon digestion by forming bonds with other phenolics, proteins, and starches found in foods [71,72,73]. While no other food enriched with aqueous and methanolic extracts from VO fruit has been reported in the literature, a study comparing VO extracts obtained with different extraction methods reported that the methanolic extract had higher antioxidant capacity than the aqueous extract [49]. In our research, we also found that antioxidant capacity increased after enrichment and food processing.

### 3.4. Biological Properties

Fruits, their extracts, and enriched foods prepared with these extracts may contain various antioxidants characterized by different mechanisms of action. Therefore, multiple methods are required to assess the antioxidant capacity of the studied substance. 2,2-diphenyl-1-picrylhydrazyl (DPPH), 2,2’-azinobis-(3-ethylbenzothiazoline-6-sulfonic acid) (ABTS), OH radical scavenging activity, ferric reducing antioxidant power (FRAP), and iron chelating power (CHP) are commonly used and easy-to-interpret methods for this purpose [74,75].

In our study, ABTS radical scavenging activity was found to be higher in AVOE in ethanolic and PBS extracts and in MVOE in samples after in vitro digestion. The highest ABTS activity in waffles after enrichment was measured in WA3 and WA6 samples (ethanolic and PBS extracts) and in WM6 in the case of samples after digestion. DPPH activity was high in AVOE (ethanolic extract) and in MVOE (samples after in vitro digestion), while no difference was detected between the PBS extracts. The highest DPPH activity in samples after enrichment was obtained in WM6. FRAP values were higher in MVOE in the case of ethanolic extracts and samples after digestion, while no difference was observed among PBS extracts. The highest FRAP value in waffles after enrichment was determined in the WM6 sample. Similarly, the highest CHP activity in samples after enrichment was again obtained in the WM6 sample. These differences can be explained by the synergy of compounds extractable with PBS-buffer solvent and compounds released by in vitro digestion and are consistent with the literature [76]. In general, enrichment with studied VO extracts increased ABTS, DPPH, FRAP, and CHP values, while decreasing OH scavenging activity. These results are consistent with the study of Gawlik-Dziki et al. [62]. Polyphenols alone may have low bioavailability, but this is offset by synergistic effects within the plant matrix. Therefore, functional/enriched foods offer advantages over single compounds. The increased activity seen in our study may also be due to the extraction method used.

Polyphenols exhibit biological activity not only as antioxidants but also by inhibiting enzymes involved in inflammation, such as lipoxygenase (LOX) and xanthine oxidase (XO) [77]. This inhibition confers potential anti-inflammatory properties on foods [78]. In our study, a significant anti-inflammatory effect was observed, particularly in waffles containing MVOE and in MVOE after in vitro digestion. This finding is consistent with the stronger effects of methanolic extracts than aqueous extracts reported in the literature [65,68,79]. This effect is thought to be due to changes in free, conjugated, and bound phenolic forms during the final stages of digestion [80]. Many of the identified phenolics have documented bioactivities that align with our functional assays: chlorogenic and caffeic acids (antioxidant, anti-inflammatory), catechin and procyanidins (strong radical scavengers and enzyme inhibitors), and tricin (anti-inflammatory and antiproliferative); thus, the compound profile detected by LC-ESI-MS provides mechanistic support for the antioxidant and anti-inflammatory activities observed in the MVOE-enriched waffles [81,82]. Several experimental studies report potential pro-inflammatory effects of dietary maltodextrin. Therefore, because maltodextrin can both act as a useful carrier/stabilizer and potentially modulate inflammatory responses, we included a dedicated maltodextrin control (CW2) to separate carrier effects from extract effects [83,84]. In our analyses no meaningful differences in anti-inflammatory properties were detected between CW1 and CW2, suggesting that the presence of maltodextrin in the control formulation did not confound the anti-inflammatory assessment. Therefore, for future placebo-controlled clinical trials, either control formulation appears suitable as a comparator; CW2 was introduced primarily as a precautionary measure to ensure that any observed effects in extract-enriched groups could be attributed to VO polyphenols rather than to the carrier.

Studies on enriched foods show that phenolic content and antioxidant capacity increase as the level of addition increases. Our findings are consistent with this literature. The release and interactions of compounds found in foods after in vitro digestion further accentuated this increase. While both types of VOFEs exhibited high phenolic content, methanolic extracts and waffles prepared with them achieved the highest antioxidant and anti-inflammatory capacity after digestion.

## 4. Materials and Methods

### 4.1. Plant Material

The *Viburnum opulus* L. (VO) fruits used in this study were obtained in September 2021 from a local grower in the Yahyalı region of Kayseri, Turkey. This area represents a transition zone to the high plateaus of Central Anatolia and is characterized by a continental climate. The growing sites typically have moderately organic, calcareous, and well-drained soils. The gilaburu plant grows naturally in the region and thrives in shrublands known for their resistance to cold climatic conditions. The bundle, including the fruits and leaves, had previously been taxonomically identified, and the results were presented in our earlier study and recorded in the Herbarium of Marmara University Faculty of Pharmacy [13]. About 1.1 kg of VO fruits was freeze-dried for 48 h in a lyophilizer at a temperature of −80 °C and a pressure of 0.045 mbar (LABCONCO, Kansas City, MO, USA). Then, seeds (~160 g) were removed from the dried fruits, and the remainder was ground into powder (MRC GRINDING MACHINE, SM-450, Holon, Israel).

### 4.2. Preparation of the Powdered VO Fruit Extracts

#### 4.2.1. Aqueous *Viburnum opulus* L. Extract (AVOE)

The powdered freeze-dried fruit (80 g) was mixed with boiling distilled water (1600 mL), boiled for 5 min, and then kept at room temperature for 15 min. After centrifugation (9000× *g*), the obtained supernatant was filtered. The extract was then freeze-dried for 24 h (as in Section 4.1) and stored in a fridge at +4 °C until use [70].

#### 4.2.2. Methanolic *Viburnum opulus* L. Extract (MVOE)

The powdered freeze-dried fruit (80 g) was first subjected to repeated macerations with *n*-hexane (total 1700 mL) for 7 × 24 h at room temperature until the residue became colorless to remove nonpolar compounds such as lipids and chlorophylls. The *n*-hexane extracts were discarded. The defatted residue was then extracted several times with methanol (a total of 2000 mL) for 8 × 24 h at room temperature until colorless to obtain the methanolic extract. The methanolic extract was evaporated using a rotary evaporator (Heidolph, Schwabach, Germany, No: 569-00100-00-0, 2012). The extract was stored in a fridge at 4 °C until use [14].

### 4.3. Characterization of the AVOE and MVOE

#### 4.3.1. Liquid Chromatography–Electrospray Ionization–Tandem Mass Spectrometry (LC–ESI–MS/MS)-Based Qualitative Analysis of Phenolic Compounds

Before LC-ESI-MS/MS analysis, both aqueous (AVOE) and methanolic (MVOE) *Viburnum opulus* L. fruit extracts were dissolved in methanol (HPLC grade) to a final concentration of 1 mg/mL, filtered through a 0.22 μm PTFE syringe filter, and injected into the LC-MS system. Polyphenolic components of the fruit extracts were identified using LC-ESI-tandem MS. Polyphenolic compounds were separated and analyzed using an Agilent 6530 with an Agilent Poroshell C18 (3 × 150 mm, 2.7 μm) analytical column maintained at 30 °C. Electrospray ionization in the negative ion mode generated complete mass and fragmentation spectra of polyphenols using a quadrupole time-of-flight analyser. Nitrogen was used as the nebulising gas and helium as the collision gas. The mobile phase content and composition followed the protocol previously reported [85].

#### 4.3.2. Preparation of Liquid Extracts from the AVOE and MVOE

The extraction was performed using two commonly used solvents: 50% ethanol containing 0.1% HCl and phosphate-buffered saline (PBS, pH ~7.4). Lyophilized extract (2 g) was homogenized with 15 mL of the extraction solvent for 2 min, shaken for 30 min (100 rpm, Multi Bio RS-24, Biosan, Riga, Latvia), and centrifuged at 9000× *g* for 15 min at 4 °C. The extraction procedure was repeated three times, and the combined supernatants were adjusted to a final volume of 50 mL. The obtained liquid extracts were used for the determination of phenolic compounds and the evaluation of biological properties.

#### 4.3.3. In Vitro Gastrointestinal Digestion (GID) of AVOE and MVOE

The lyophilized extracts were subjected to GID according to the method previously described by Durak et al. [86]. The samples (2 g) were homogenized in a Stomacher Laboratory Blender for 1 min to simulate mastication in the presence of 5 mL of a simulated saliva solution containing 7 mM NaHCO3, 0.35 mM NaCl (pH 6.75), and α-amylase (E.C. 3.2.1.1., 200 U per mL of enzyme activity). Subsequently, the mixture was stirred for 10 min at 37 °C in the dark. For gastric digestion, the solution was adjusted to pH 2.5 with 1 M HCl, and then 15 mL of 300 U/mL of pepsin (from porcine stomach mucosa, pepsin A, EC 3.4.23.1) in 0.03 mol/L NaCl, pH −1.2, was added. The reaction was carried out for 60 min at 37 °C. Next, the solution was adjusted to pH 7 with 1 M NaOH, and then 15 mL of a mixture of a 0.7% pancreatin solution and a 2.5% bile extract solution were added. The incubation was carried out for 120 min at 37 °C in darkness. Next, the samples were centrifuged, and the supernatants were collected. The obtained digestion fluids were used for the determination of phenolic compounds and the evaluation of biological properties.

#### 4.3.4. Determination of Phenolic Compounds in Aqueous–Ethanolic and PBS Extracts and in GID Samples

Total phenolic content (TPC) was measured according to a previously described method [87] adopted for a microplate reader (Epoch 2 Microplate Spectrophotometer, BioTek Instruments, Winooski, VT, USA). A total of 10 microliters of extract were mixed with 10 µL of water and 40 µL of Folin–Ciocalteau reagent diluted in water at a 1:5 ratio. After 3 min, 250 µL of 10% sodium carbonate was added, and the solution was thoroughly mixed. A 50% ethanol or digested control (H_2_O) sample was used as the standard. The absorbance was measured at 725 nm after 30 min of incubation and normalized against the standard. Results were expressed as mg gallic acid equivalents (GAE) per gram of dry weight (DW). The analyses were performed in 4 replicates.

Total flavonoid content (TFC) was determined according to a previously described method [88] adopted for a microplate reader (Epoch 2 Microplate Spectrophotometer, BioTek Instruments, Winooski, VT, USA) and expressed as mg quercetin equivalents (QE) per g DW. A total of 100 µL of extract was mixed with 100 µL of 2% (*w*/*v*) AlCl_3x_6H_2_O solution (in methanol) and incubated at room temperature for 10 min. Thereafter, absorbance at 430 nm was measured. The analyses were performed in 4 replicates.

Phenolic acid content (PAC) was estimated using the Arnov method [89] adopted for a microplate reader (Epoch 2 Microplate Spectrophotometer, BioTek Instruments, Winooski, VT, USA) and expressed as µg caffeic acid equivalents (CAE) per g DW. A total of 10 µL of sample was mixed with 60 µL of distilled water, 10 µL 0.5 M HCl, 10 µL of Arnov reagent (10 g sodium molybdate and 10 g sodium nitrite dissolved in 100 mL of distilled water), and 10 µL 1 M NaOH. Absorbance was measured at 490 nm. The analyses were performed in 4 replicates.

#### 4.3.5. Evaluation of Antioxidant Activities of the Aqueous–Ethanolic and PBS Extracts and GID Samples

##### Antiradical Activities

The previously described procedure [90] was used for determination of (2,2′-azino-bis(3-ethylbenzothiazoline-6-sulfonic acid)) radical (ABTS^•+^) scavenging activity. 2,2-Diphenyl-1-picrylhydrazyl radical (DPPH*) scavenging activity was analyzed according to [91]. Hydroxyl radical (OH*) scavenging activity was measured using the Fenton reaction method (Fe_2_SO_4_ + H_2_O_2_) [92].

For the ABTS assay, ABTS radical cations (ABTS^+•^) were produced by reacting 7 mM stock solution of ABTS with 2.45 mM potassium persulfate (final concentration) and allowing the mixture to stand in the dark for at least 6 h at room temperature before use. The ABTS^+•^ solution was diluted to an absorbance of 0.7 ± 0.05 at 734 nm (Epoch 2 Microplate Spectrophotometer, BioTek Instruments, Winooski, VT, USA). A total of 10 µL of the sample was added to 250 µL of ABTS^+•^. The absorbance at 734 nm was measured both at the beginning and after 2.5 min of reaction against a blank sample.

For DPPH assay, 250 µL of DPPH solution was mixed with 10 µL of the sample and measured at 517 nm using a UV/Vis microplate spectrophotometer (BioTek, Model Epoch2TC, Winooski, VT, USA) after 15 min of incubation in room temperature.

In case of determination of hydroxyl radical scavenging activity, OH* were generated by the Fenton reaction in the system of FeSO_4_ and H_2_O_2_. The reaction mixture consisted of 25 µL FeSO_4_ (8 mM), 40 µL H_2_O_2_ (6 mM), 25 µL distilled water, 50 µL of the sample, and 10 µL sodium salicylate (20 mM). The total mixture was incubated at 37 °C for 1 h, and then the absorbance of the mixture was recorded at 562 nm using a UV/Vis microplate spectrophotometer (BioTek, Model Epoch2TC, Winooski, VT, USA).

All results of antiradical activities were expressed as Trolox equivalents per g of DW (mg TE/g DW). The analyses were performed in 4 replicates.

##### Fe^2+^ Chelating Power (CHP) and Ferric Reducing Antioxidant Power (FRAP)

CHP ability was measured according to [93] and expressed as µg ethylenediaminetetraacetic acid (EDTA) equivalents per g DW.

The samples (100 µL) were added to 4 µL of 2 mM FeCl_2_ solution and 4 µL 5 mM ferrozine, and the mixture was shaken vigorously and left standing at room temperature for 10 min. Then, the absorbance of the solution was measured spectrophotometrically at 562 nm (BioTek, Model Epoch2TC, Winooski, VT, USA). The control sample contained 100 µL of solvent (PBS or ethanol), 4 µL 2 mM FeCl_2_ solution and 4 µL 5 mM ferrozine. The percentage of inhibition of ferrozine—Fe^2+^ complex formation was calculated using the formula:

% inhibition = (1 − A_A_/A_C_) × 100

where:

A_C_ is absorbance of the control (solvent instead extract) and A_A_ is absorbance of the sample.

In the assessment of the FRAP of studied samples, the reaction mixture containing 50 µL of sample and phosphate buffer (50 µL, 200 mmol/L, pH 6.6) and 50 µL of a 1 g/100 mL aqueous solution of potassium ferricyanide K_3_[Fe(CN_6_)] was shaken and incubated at 50 °C for 20 min. A total of 50 µL of trichloroacetic acid (10 g/100 mL) was added to the mixture, which was then centrifuged at 25× *g* for 10 min. The upper layer of the solution (100 µL) was mixed with distilled water (100 µL) and 40 µL of FeCl_3_ (0.1 g/100 mL). Then, the absorbance was measured at 700 nm (BioTek, Model Epoch2TC, Winooski, VT, USA). FRAP was determined according to [94] and expressed as mg TE/g DW. The analyses were performed in 4 replicates.

#### 4.3.6. Evaluation of Anti-Inflammatory Activity of the Aqueous–Ethanolic and PBS Extracts, and in Digestion Fluids

Lipoxygenase (LOX) and xanthine oxidase (XO) inhibitory activities were determined as described in earlier studies [95,96] and are expressed as the concentration of sample required to inhibit 50% of enzyme activity (EC_50_, mg DW/mL). The analyses were performed in 4 replicates.

Lipoxygenase activity was determined spectrophotometrically at a temperature of 25 °C by measuring the increase in absorbance at 234 nm over a 3 min period. The reaction mixture contained 234 µL of 1/15 M phosphate buffer with pH 7.0, 10 µL of lipoxygenase solution (Sigma, L7395, Poznań, Poland) (167 U mL^−1^), and different amounts of inhibitor (studied sample) solution (10 µL, 20 µL, 30 µL). After pre-incubation of the mixture for 3 min, the reaction was initiated by adding 20 µL 2.5 mmol L^−1^ linoleic acid. One unit of LOX activity was defined as an increase in absorbance of 0.001 per minute at 234 nm. The corresponding control contains the same concentration of enzyme in the absence of an inhibitor.

For XO activity determination, 30 µL of the sample was diluted in 110 µL of 1/15 M/L phosphate buffer (pH 7.5) and 20 µL of enzyme solution (Sigma, X1875) (0.01 U/mL in 1/15 M/L phosphate buffer). After preincubation at 30 °C for 10 min, the reaction was started by adding 140 µL of 0.15 mM/L xanthine solution. The absorbance (295 nm) was measured every minute for 3 min. The corresponding control contains the same concentration of enzymes in the absence of an inhibitor.

### 4.4. Preparation of Waffles

Two controls (CW1 and CW2) and three AVO-enriched (WA1, WA3, WA6) and three MVO-enriched (WM1, WM3, WM6) waffle groups were produced. The base waffle formulation (CW1) contained, per 100 g of batter, 43 g wheat flour, 57 g water, 1.4 g sunflower oil, 0.7 g baking powder, 1.4 g vanilla sugar, and 3.6 g egg whites. In the CW2 waffle recipe, water was replaced with 6 g maltodextrin. In the improved waffles, water was replaced with increasing levels (1, 3, or 6 g) of aqueous (WA1, WA3, WA6) or methanolic (WM1, WM3, WM6) *Viburnum opulus* L. fruit extracts, respectively. Extract incorporation levels were based on a previous clinical study [18]. All ingredient quantities are expressed in grams, including water and sunflower oil, to ensure uniform measurement across samples using a single scale and maintain consistency during batter preparation and calibration. The formulation components were mixed using a household electric mixer at medium speed for 3 min until a homogeneous batter was obtained. Approximately 70 g of batter was poured into each waffle cavity of a double-plate electric waffle maker (Tefal, Rumilly, France), allowing two waffles to be baked simultaneously per batch. The waffles were baked at 180 °C for 11 min, cooled to room temperature, vacuum-packed, and stored at −20 °C until analysis.

### 4.5. Analyses of Physicochemical and Sensory Properties of the Waffles

For physicochemical and sensory properties of the waffles, fresh samples (i.e., cooled to room temperature) were used.

#### 4.5.1. Water Absorption Capacity (WAC)

WAC of the waffles was determined following [97], with modifications [98] as follows: each 0.5 g sample was added to 15 mL of distilled water and stirred for 5 min using a magnetic stirrer. The suspension was then centrifuged at 5000× *g* for 30 min, and the supernatant was measured in a 10 mL graduated cylinder. WAC was determined as the difference between the initial volume of water added to the sample and the measured volume of the supernatant after centrifugation. The analyses were performed in 4 replicates.

#### 4.5.2. Oil Absorption Capacity (OAC)

The OAC of the waffles was determined following [97], with modifications [32]. One gram of the sample was mixed with 5 mL of oil in a centrifuge tube and allowed to stand at room temperature for 30 min. It was then centrifuged at 15,000× *g* for 15 min. The volume of oil in the sediment was measured. OAC was calculated as milliliters of oil absorbed per gram of sample. The analyses were performed in 4 replicates.

#### 4.5.3. Surface Color Analysis

The color parameters were measured using a colorimeter (NH310, 3nh, Guangzhou, China). The CIE color values L* (lightness), a* (redness), and b* (yellowness) were measured to describe the color of the waffles. The measuring aperture diameter was 8 mm. The colorimeter was calibrated using the standard of white (L* = 95.82; a* = −0.44; b* = 2.5) [99]. Color analyses were performed in 4 replicates.

#### 4.5.4. Texture Assessment

Cutting force (CF, maximum force in kg), work of shear (WS, the area under the force–time curve, in kg·s), and puncture strength (PS, maximum force in MPa) of waffle samples (62 × 77 mm) taken from the midsection were measured using a TA.XT.PLUS Texture Analyzer (Stable Micro Systems, Godalming, UK), following a method described in an earlier study [98]. A knife-edge with a slotted blade insert (HDP/BS set) was used for CF and WS determination. Pre-test and test speeds were set to 1.5 mm/s and 10 mm/s, respectively. For the PS test, a cylindrical probe (P/3) with a diameter of 3 mm was used. The penetration test was performed in the central depressions of the waffle surface. The PS was calculated by Equation (1):

PS = F/A(1)

where F is the maximum force (N) and A is the cross-sectional area of the single depressions in a multi-dimple waffle structure (thickness × length of the dimple, mm^2^).

The thickness of the waffles in the dimples was measured using a digital caliper. All texture analyses were performed in nine replicates, using samples taken from different waffles.

#### 4.5.5. Sensory Analysis

Sensory attributes (taste, odor, texture, appearance, overall impression) of the fresh waffles were evaluated by 12 panelists aged 20–50 years. Although the panelists had no formal sensory training, they received brief standardized instructions on how to evaluate each sample and were asked to rinse their mouths with water between tastings. Each panelist received one small square from each of the 8 waffle samples (each waffle consisting of 16 squares), coded with random three-digit numbers to ensure sample blinding. Evaluation of the samples was performed using a 9-point hedonic scale (1 = poor, 9 = excellent) [52].

### 4.6. Analyses of Biological Properties of the Waffles

#### 4.6.1. Preparation of Liquid Extracts from the Waffles

Waffles were freeze-dried (as in Section 4.1) and ground using a laboratory mill (SM-450C, MRC, Warsaw, Poland). Extractions with aqueous–ethanolic solvent and PBS were carried out according to the procedure outlined in Section 4.3.2.

#### 4.6.2. In Vitro Gastrointestinal Digestion (GID) of the Waffles

The GID of powdered freeze-dried waffles was carried out according to the procedure outlined in Section 4.3.3.

#### 4.6.3. Determination of Phenolic Compounds, Antioxidant, and Anti-Inflammatory Activities of Aqueous–Ethanolic and PBS Extracts and in GID Samples of Waffles

The methodologies outlined in Section 4.3.4, Section 4.3.5 and Section 4.3.6 were employed to evaluate phenolic content as well as antioxidant and anti-inflammatory activities in the samples prepared in Section 4.6.1 and Section 4.6.2

### 4.7. Comparison of Total Antioxidant Capacity of the AVOE, MVOE, and Waffles

Antioxidant ranking of the samples was performed according to an earlier study [100]. The samples were ranked according to their TPC, TFC, PAC, antiadical, CHP, and FRAP values. For each parameter, the highest value was given the lowest rank, after which the total and mean ranks were calculated. The sample with the lowest total rank was considered to have the best overall antioxidant capacity.

### 4.8. Statistical Analysis

Statistical analysis was performed using Statistica version 13.3 (StatSoft, Kraków, Poland). Significant differences at the level of *p* < 0.05 between the samples were determined using one-way ANOVA followed by post hoc Tukey’s HSD test.

## 5. Conclusions

In conclusion, *Viburnum opulus* L. extracts can be utilized as functional food ingredients to enhance the nutritional value and promote health benefits. Phenolic content and antioxidant capacity increased with increasing addition levels, and this effect became more pronounced after in vitro digestion. Both types of VO fruit extracts and the waffles prepared with them exhibited high phenolic content, and the antioxidant and anti-inflammatory capacity of the MVOE and the corresponding waffles were found to be higher than those of the other groups. These findings demonstrate that the functional properties of VO extract-enriched waffles were preserved, with formulation WM6 exhibiting the best performance. These results indicate that *Viburnum opulus* L. fruit extracts can be used as functional food ingredients to increase nutritional value and enhance health-promoting properties and can be considered a promising resource. The findings demonstrate that the functional properties of VO extract-enriched foods can be preserved, highlighting their potential as a functional product that can form the basis for future studies on healthy individuals or clinical trials.

## Figures and Tables

**Table 1:** Phenolic compounds identified by LC-ESI-tandem MS in aqueous VO fruit extract.

Rt (min)	[M-H]- (*m*/*z*)	Fragment(s) Ions *m*/*z* (MS Mode)	Compound	Refs.
2.300	133	115, 71	malic acid	st.
2.267	191	127, 85	quinic acid	st.
5.774	354	191	chlorogenic acid	st.
6.023	709	354, 192	chlorogenic acid dimer	st.
9.828	578	426, 353, 290, 181, 125	prosiyanidin b2	[46].
10.490	330	286, 240, 171	tricin	[47].
10.680	289	245, 205	catechin	st.
12.500	452	342, 300, 89, 105	cinchonain	[45].

Rt—retention time, st.—standard.

**Table 2:** Phenolic compounds identified by LC-ESI-tandem MS in methanolic VO fruit extract.

Rt (min)	[M-H]- (*m*/*z*)	Fragment(s) Ions *m*/*z* (MS Mode)	Compound	Refs.
2.193	133	115, 89, 71, 59	malic acid	st.
2.523	191	85	quinic acid	st.
6.279	354	191	chlorogenic acid	st.
10.606	289	245, 109	catechin	st.
10.714	452	395, 341, 290, 260	cinchonain	[45].

Rt—retention time, st.—standard.

**Table 3:** Physicochemical properties of wheat waffles.

Parameters	CW1	CW2	WA1	WA3	WA6	WM1	WM3	WM6
L*	65.34 ± 1.16 ^a^	69.01 ± 2.76 ^a^	65.90 ± 1.96 ^a^	64.81 ± 0.06 ^a^	63.36 ± 2.40 ^a^	36.83 ± 0.58 ^b^	35.00 ± 0.97 ^c^	29.19 ± 0.34 ^d^
a*	0.41 ± 0.09 ^a^	1.31 ± 0.48 ^a^	2.01 ± 0.30 ^ab^	2.29 ± 0.38 ^ab^	4.32 ± 0.41 ^c^	2.57 ± 0.23 ^ab^	5.06 ± 0.53 ^c^	6.40 ± 0.16 ^d^
b*	3.41 ± 0.29 ^a^	12.84 ± 1.33 ^b^	10.42 ± 0.35 ^c^	10.94 ± 0.48 ^bc^	13.91 ± 1.21 ^bd^	5.62 ± 0.55 ^e^	8.50 ± 0.30c ^ef^	6.80 ± 0.18 ^e^
CF (kg)	6.4 ± 0.27 ^a^	6.09 ± 0.52 ^a^	4.05 ± 0.40 ^b^	4.57 ± 0.40 ^b^	6.06 ± 0.35 ^a^	4.54 ± 0.24 ^b^	4.75 ± 0.34 ^b^	6.92 ± 0.47 ^a^
WS (kg s)	26.65 ± 2.54 ^a^	24.27 ± 2.15 ^a^	15.23 ± 1.40 ^b^	15.68 ± 1.68 ^b^	25.20 ± 1.66 ^a^	14.99 ± 1.85 ^b^	15.60 ± 1.13 ^b^	28.82 ± 2.52 ^a^
PS (MPa)	7.52 ± 0.32 ^a^	13.57 ± 0.78 ^b^	7.10 ± 0.74 ^a^	8.07 ± 0.94 ^a^	8.75 ± 1.64 ^ac^	7.05 ± 0.52 ^ad^	7.32 ± 0.57 ^a^	8.49 ± 1.23 ^a^
WAC (g/100gDW)	335.88 ± 7.17 ^ab^	362.09 ± 23.29 ^bc^	393.89 ± 10.13 ^c^	333.07 ± 3.01 ^ab^	322.43 ± 21.64 ^ab^	396.23 ± 4.94 ^c^	334.62 ± 8.35 ^ab^	328.84 ± 5.66 ^ab^
OAC (g/gDW)	252.30 ± 9.59 ^a^	255.94 ± 13.41 ^a^	256.38 ± 7.20 ^a^	259.31 ± 18.61 ^a^	250.73 ± 18.93 ^a^	226.16 ± 0.81 ^a^	235.43 ± 5.00 ^a^	242.33 ± 1.03 ^a^

CW1: control waffles with white flour; CW2: control waffles with maltodextrin; WA1, 3, and 6: waffles with *Viburnum opulus* L. aqueous extract; WM1, 3, and 6: waffles with *Viburnum opulus* L. metanolic extract; L*: lightness (0 = black, 100 = white); a*: green (–) to red (+) axis; b*: blue (–) to yellow (+) axis; CF—cutting force; WS—work of shear; PS—puncture strength; WAC—water absorption capacity; OAC—oil absorption capacity. Values in the rows denoted with different letters are significantly different (*p* < 0.05).

**Table 4:** Sensory attributes of wheat waffles.

Samples	Taste	Smell	Texture	Appearance	Overall Impression
CW1	4.33 ± 2.15 ^a^	5.33 ± 2.06 ^a^	4.67 ± 2.27 ^a^	5.83 ± 1.59 ^a^	5.17 ± 1.80 ^a^
CW2	6.50 ± 1.51 ^a^	6.50 ± 1.78 ^ab^	5.33 ± 1.83 ^a^	6.08 ± 1.88 ^a^	6.08 ± 1.78 ^ab^
WA1	4.50 ± 2.15 ^a^	5.33 ± 2.30 ^a^	4.50 ± 2.32 ^a^	4.83 ± 1.40 ^a^	4.75 ± 1.71 ^a^
WA3	4.08 ± 1.50 ^ac^	5.58 ± 1.88 ^a^	4.17 ± 1.47 ^a^	4.58 ± 1.88 ^a^	4.25 ± 1.22 ^a^
WA6	3.58 ± 1.83 ^ab^	4.50 ± 2.39 ^b^	4.00 ± 2.00 ^a^	4.58 ± 1.97 ^a^	4.08 ± 1.83 ^b^
WM1	5.92 ± 1.83 ^a^	5.92 ± 2.02 ^a^	5.42 ± 1.56 ^a^	5.75 ± 1.96 ^a^	5.75 ± 1.87 ^a^
WM3	6.42 ± 1.98 ^ad^	6.33 ± 2.10 ^a^	5.50 ± 2.11 ^a^	5.83 ± 1.95 ^a^	6.17 ± 1.90 ^a^
WM6	6.42 ± 2.46 ^ad^	6.33 ± 2.64 ^a^	5.92 ± 2.23 ^a^	5.92 ± 2.28 ^a^	6.08 ± 2.54 ^a^

CW1: control waffles with white flour; CW2: control waffles with maltodextrin; WA1, 3, and 6: waffles with *Viburnum opulus* L. aqueous extract; WM1, 3, and 6: waffles with *Viburnum opulus* L. metanolic extract. Values in the columns denoted with different letters are significantly different (*p* < 0.05).

**Table 5:** Content of phenolic compounds in waffles and VO extracts.

Samples	Ethanolic			PBS			GID		
	TPC [mg GAE/gDW]	TFC [mgQE/gDW]	PAC [µg CAE/gDW]	TPC [mg GAE/gDW]	TFC [mgQE/gDW]	PAC [µg CAE/gDW]	TPC [mg GAE/gDW]	TFC [mgQE/gDW]	PAC [µg CAE/gDW]
CW1	1.29 ± 0.08 ^a^	2.09 ± 0.07 ^a^	6.85 ± 1.29 ^a^	0.81 ± 0.06 ^a^	2.64 ± 0.33 ^a^	4.99 ± 0.15 ^a^	2.56 ± 0.20 ^a^	22.96 ± 2.94 ^ab^	5.21 ± 0.18 ^a^
CW2	3.55 ± 0.53 ^bc^	4.11 ± 0.82 ^ab^	5.85 ± 0.13 ^a^	1.71 ± 0.46 ^ab^	3.02 ± 0.51 ^a^	5.39 ± 0.21 ^a^	3.27 ± 0.33 ^a^	18.17 ± 4.89 ^a^	7.36 ± 0.42 ^a^
WA1	1.47 ± 0.07 ^a^	2.55 ± 0.33 ^ab^	5.03 ± 0.18 ^a^	1.05 ± 0.05 ^ab^	2.96 ± 0.29 ^a^	5.10 ± 0.14 ^a^	5.77 ± 0.38 ^ab^	47.60 ± 8.85 ^abc^	7.41 ± 0.35 ^a^
WA3	3.05 ± 0.17 ^b^	3.05 ± 0.15 ^ab^	5.85 ± 0.20 ^a^	2.08 ± 0.16 ^ab^	3.50 ± 0.38 ^ab^	5.71 ± 0.51 ^a^	6.29 ± 0.37 ^ab^	56.12 ± 3.17 ^abc^	7.50 ± 0.40 ^a^
WA6	4.72 ± 0.09 ^cd^	3.74 ± 0.19 ^ab^	8.14 ± 0.39 ^a^	3.00 ± 0.31 ^bc^	3.82 ± 0.27 ^ab^	17.01 ± 2.99 ^c^	6.68 ± 0.36 ^ab^	65.02 ± 4.46 ^bc^	7.65 ± 0.41 ^a^
WM1	4.93 ± 1.48 ^d^	4.45 ± 0.17 ^b^	6.44 ± 0.33 ^a^	2.63 ± 1.14 ^abc^	4.91 ± 0.88 ^a^	6.58 ± 0.67 ^a^	14.38 ± 0.12 ^bc^	32.65 ± 6.94 ^abc^	9.89 ± 0.27 ^ab^
WM3	5.81 ± 0.55 ^d^	8.48 ± 1.68 ^c^	9.62 ± 0.21 ^a^	4.31 ± 0.53 ^cd^	5.72 ± 0.92 ^cd^	12.20 ± 0.99 ^b^	15.25 ± 0.16 ^bc^	47.76 ± 7.83 ^abc^	17.26 ± 1.88 ^ab^
WM6	7.58 ± 0.68 ^e^	14.50 ± 1.98 ^e^	10.89 ± 0.19 ^a^	5.78 ± 0.79 ^de^	7.36 ± 0.46 ^de^	14.93 ± 2.23 ^bc^	17.47 ± 1.01 ^c^	63.34 ± 0.82 ^c^	22.19 ± 4.27 ^b^
AVOE	11.38 ± 0.47 ^f^	4.63 ± 0.12 ^b^	142.31 ± 11.60 ^c^	7.11 ± 0.90 ^e^	42.19 ± 2.02 ^f^	69.90 ± 2.61 ^e^	94.41 ± 13.86 ^d^	44.51 ± 18.07 ^abc^	44.48 ± 2.04 ^c^
MVOE	10.39 ± 0.70 ^f^	11.24 ± 0.22 ^d^	30.60 ± 1.76 ^b^	11.87 ± 1.83 ^f^	7.98 ± 1.03 ^e^	42.17 ± 4.50 ^d^	122.59 ± 6.91 ^e^	464.15 ± 47.55 ^d^	231.56 ± 21.51 ^d^

CW1: control waffles with white flour; CW2: control waffles with maltodextrin; WA1, 3, and 6: waffles with *Viburnum opulus* L. aqueous extract; WM1, 3, and 6: waffles with *Viburnum opulus* L. metanolic extract; AVOE: *Viburnum opulus* L. aqueous extract; MVOE: *Viburnum opulus* L. metanolic extract; TPC: total phenolic compounds; TFC: total flavonoid content; PAC: phenolic acid content. Values in the columns denoted with different letters are significantly different (*p* < 0.05).

**Table 6:** The antioxidative activities of waffles and VO extracts.

Ethanolic					
Samples	ABTS (mg TE/gDW)	DPPH (mg TE/gDW)	OH Radical (mg TE/gDW)	FRAP (mg TE/gDW)	CHP (mg EDTA/gDW)
CW1	2.67 ± 0.69 ^a^	223.71 ± 10.32 ^bc^	39.22 ± 2.10 ^e^	2.70 ± 0.14 ^a^	171.98 ± 78.14 ^a^
CW2	1.71 ± 1.62 ^a^	1.37 ± 1.27 ^a^	19.02 ± 6.64 ^ab^	2.33 ± 0.12 ^a^	290.24 ± 37.37 ^a^
WA1	4.01 ± 0.87 ^a^	227.07 ± 8.05 ^bc^	30.00 ± 3.03 ^cde^	3.33 ± 0.14 ^a^	525.57 ± 164.09 ^b^
WA3	23.37 ± 5.97 ^bc^	251.99 ± 49.18 ^c^	31.17 ± 2.69 ^de^	22.06 ± 3.03 ^b^	971.00 ± 65.04 ^b^
WA6	49.94 ± 21.06 ^d^	220.36 ± 30.12 ^bc^	33.66 ± 3.99 ^de^	22.85 ± 3.07 ^b^	992.17 ± 101.28 ^b^
WM1	5.98 ± 3.74 ^ab^	11.85 ± 0.64 ^a^	11.64 ± 7.26 ^a^	138.05 ± 2.76 ^d^	1498.88 ± 372.11 ^c^
WM3	14.37 ± 5.04 ^abc^	36.63 ± 17.51 ^a^	12.67 ± 6.91 ^a^	151.40 ± 7.57 ^e^	1935.15 ± 130.07 ^c^
WM6	15.84 ± 2.32 ^abc^	145.04 ± 64.28 ^b^	14.89 ± 3.41 ^ab^	157.03 ± 5.79 ^e^	4835.15 ± 376.83 ^e^
AVOE	88.40 ± 3.62 ^e^	261.02 ± 8.73 ^c^	25.52 ± 2.87 ^bcd^	45.07 ± 6.10 ^c^	3905.83 ± 569.23 ^d^
MVOE	30.10 ± 2.35 ^c^	194.29 ± 13.84 ^b^	18.65 ± 1.75 ^abc^	211.63 ± 3.20 ^f^	3227.66 ± 301.96 ^d^
PBS					
CW1	6.26 ± 3.37 ^a^	4.21 ± 2.60 ^a^	33.38 ± 3.47 ^ab^	18.42 ± 1.46 ^a^	4812.51 ± 556.10 ^a^
CW2	1.00 ± 0.8 ^a^	4.44 ± 3.00 ^a^	43.08 ± 1.87 ^b^	17.33 ± 0.98 ^a^	5837.05 ± 286.17 ^b^
WA1	21.11 ± 5.39 ^a^	3.83 ± 4.44 ^a^	35.76 ± 1.89 ^ab^	18.50 ± 1.00 ^a^	6427.73 ± 7.85 ^cd^
WA3	31.01 ± 12.63 ^a^	4.18 ± 4.60 ^a^	33.61± 2.75 ^ab^	22.88 ± 3.44 ^a^	6608.35 ± 26.82 ^cde^
WA6	49.49 ± 35.43 ^a^	4.49 ± 9.37 ^a^	32.73 ± 3.00 ^a^	30.42 ± 4.41 ^a^	6698.66 ± 116.83 ^cde^
WM1	7.36 ± 0.99 ^a^	15.12 ± 1.98 ^a^	27.74 ± 5.75 ^a^	142.46 ± 1.37 ^b^	6435.58 ± 56.45 ^cd^
WM3	8.66 ± 0.40 ^a^	104.88 ± 10.33 ^b^	33.70 ± 5.70 ^ab^	149.70 ± 8.17 ^b^	6601.16 ± 157.41 ^cde^
WM6	20.65 ± 18.16 ^a^	152.47 ± 6.07 ^b^	36.05 ± 5.54 ^ab^	159.92 ± 5.23 ^b^	6963.10 ± 40.77 ^e^
AVOE	982.75 ± 611.39 ^b^	274.78 ± 8.27 ^c^	34.12 ± 5.66 ^ab^	238.85 ± 27.22 ^c^	6302.08 ± 89.19 ^bc^
MVOE	29.41 ± 1.00 ^a^	257.53 ± 35.15 ^c^	36.23 ± 2.78 ^ab^	208.07 ± 7.98 ^c^	6817.56 ± 46.91 ^de^
GID					
CW1	96.90 ± 59.29 ^a^	1.63 ± 0.33 ^a^	308.74 ± 47.65 ^a^	25.61 ± 3.86 ^a^	6.121.46 ± 53.45 ^a^
CW2	70.50 ± 13.38 ^a^	0.79 ± 0.49 ^a^	328.92 ± 23.61 ^b^	25.83 ± 2.13 ^a^	6.262.81 ± 51.89 ^ab^
WA1	113.40 ± 39.77 ^a^	1.65 ± 0.32 ^a^	361.26 ± 21.53 ^ab^	28.88 ± 2.72 ^a^	6.368.83 ± 126.30 ^abc^
WA3	299.55 ± 64.80 ^a^	2.97 ± 0.63 ^a^	363.26 ± 68.03 ^ab^	31.45 ± 2.33 ^a^	6.655.47 ± 23.56 ^c^
WA6	323.02 ± 54.12 ^a^	3.42 ± 0.85 ^a^	365.21 ± 42.80 ^ab^	39.60 ± 2.01 ^a^	11.110.26 ± 21.31 ^e^
WM1	354.73 ± 50.05 ^a^	101.75 ± 29.43 ^c^	381.40 ± 41.63 ^ab^	212.86 ± 7.05 ^c^	11.505.84 ± 318.11 ^f^
WM3	371.57 ± 9.59 ^a^	133.62 ± 24.89 ^cd^	383.09 ± 46.59 ^ab^	238.68 ± 13.94 ^d^	11.675.38 ± 26.10 ^f^
WM6	432.47 ± 18.68 ^a^	162.00 ± 5.28 ^d^	401.19 ± 98.20 ^ab^	260.08 ± 5.32 ^e^	11.686.68 ± 124.50 ^f^
AVOE	1158.36 ± 614.25 ^c^	23.38 ± 0.39 ^b^	368.92 ± 23.60 ^ab^	165.84 ± 11.16 ^b^	6.608.35 ± 65.86 ^bc^
MVOE	5266.10 ± 1.444.89 ^b^	412.64 ± 29.38 ^e^	436.92 ± 34.15 ^b^	318.18 ± 7.70 ^f^	9.403.60 ± 275.51 ^d^

CW1: control waffles with white flour; CW2: control waffles with maltodextrin; WA1, 3, and 6: waffles with *Viburnum opulus* L. aqueous extract; WM1, 3, and 6: waffles with *Viburnum opulus* L. metanolic extract; AVOE: *Viburnum opulus* L. aqueous extract; MVOE: *Viburnum opulus* L. metanolic extract; ABTS: radical scavenging ability against ABTS^+•^; DPPH: radical scavenging ability against DPPH^•^, OH radical—scavenging ability against OH^•^, FRAP: ferric reducing power, CHP: chelating power. Values in the columns denoted with different letters are significantly different (*p* < 0.05).

**Table 7:** Potential anti-inflammatory activities of waffles and VO extracts.

Samples	Ethanolic		PBS		GID	
	İnhibition of LOX EC_50_ [mgDW/mL]	İnhibition of XO EC_50_ [mgDW/mL]	İnhibition of LOX EC_50_ [mgDW/mL]	İnhibition of XO EC_50_ [mgDW/mL]	İnhibition of LOX EC_50_ [mgDW/mL]	İnhibition of XO EC_50_ [mgDW/mL]
CW1	20.88 ± 0.94 ^d^	14.47 ± 0.11 ^cd^	21.71 ± 2.60 ^e^	20.59 ± 0.04 ^h^	18.51 ± 1.44 ^f^	29.15 ± 0.85 ^f^
CW2	17.35 ± 0.75 ^c^	14.74 ± 0.11 ^d^	21.93 ± 0.89 ^e^	20.35 ± 0.28 ^gh^	16.17 ± 1.51 ^ef^	28.22 ± 0.65 ^f^
WA1	17.20 ± 0.60 ^c^	14.13 ± 0.13 ^c^	20.65 ± 1.10 ^de^	19.77 ± 0.06 ^g^	13.98 ± 1.10 ^de^	25.64 ± 0.70 ^e^
WA3	16.57 ± 1.79 ^bc^	13.98 ± 0.09 ^c^	17.83 ± 2.54 ^cd^	17.01 ± 0.36 ^f^	13.24 ± 3.23 ^cde^	19.36 ± 0.23 ^d^
WA6	15.16 ± 0.97 ^abc^	12.95 ± 0.17 ^b^	14.63 ± 1.05 ^bc^	14.61 ± 0.09 ^e^	11.95 ± 0.23 ^bcd^	10.70 ± 0.26 ^c^
WM1	14.67 ± 0.78 ^ab^	14.39 ± 0.29 ^cd^	14.20 ± 0.54 ^bc^	14.30 ± 0.01 ^de^	8.76 ± 0.33 ^b^	9.83 ± 0.10 ^bc^
WM3	14.45 ± 0.73 ^ab^	14.01 ± 0.34 ^c^	14.30 ± 0.08 ^bc^	13.34 ± 0.43 ^c^	8.34 ± 0.06 ^b^	9.44 ± 0.09 ^b^
WM6	14.14 ± 0.14 ^ab^	14.08 ± 0.20 ^c^	12.91± 0.53 ^ab^	13.93 ± 0.04 ^cd^	8.55 ± 0.16 ^b^	9.25 ± 0.01 ^b^
AVOE	15.82 ± 0.58 ^abc^	13.89 ± 0.24 ^c^	10.19 ± 0.07 ^a^	9.68 ± 0.02 ^a^	9.84 ± 0.16 ^bc^	9.99 ± 0.10 ^bc^
MVOE	13.40 ± 0.08 ^a^	12.26 ± 0.27 ^a^	12.20 ± 0.14 ^ab^	11.43 ± 0.14 ^b^	4.03 ± 0.11 ^a^	6.45 ± 0.05 ^a^

CW1: control waffles with white flour; CW2: control waffles with maltodextrin; WA1, 3, and 6: waffles with *Viburnum opulus* L. aqueous extract; WM1, 3, and 6: waffles with *Viburnum opulus* L. metanolic extract; AVOE: *Viburnum opulus* L. aqueous extract; MVOE: *Viburnum opulus* L. metanolic extract. Values in the columns denoted with different letters are significantly different (*p* < 0.05).

**Table 8:** Ranking of antioxidant capacity of samples after in vitro digestion of the studied waffles and VO extracts.

	Ranking of Antioxidant Capacity ^1^								Total Rank	Mean Rank	Gen. Performance ^2^
	TPC	FCA	PAC	ABTS	DPPH	OH	FRAP	CHP			
CW1	10	9	10	9	9	10	10	10	77	9.63	10
CW2	9	10	9	10	10	9	9	9	75	9.38	9
WA1	8	6	8	8	8	8	8	8	62	7.75	8
WA3	7	4	7	7	7	7	7	6	52	6.5	7
WA6	6	2	6	6	6	6	6	5	43	5.38	6
WM1	5	8	5	5	4	4	4	4	39	4.88	5
WM3	4	5	4	4	3	3	3	3	29	3.63	3
WM6	3	3	3	3	2	2	2	2	20	2.5	2
AVOE	2	7	2	2	5	5	5	7	35	4	4
MVOE	1	1	1	1	1	1	1	1	8	1	1

CW1: control waffles with white flour; CW2: control waffles with maltodextrin; WA1, 3, and 6: waffles with *Viburnum opulus* L. aqueous extract; WM1, 3 and 6: waffles with *Viburnum opulus* L. metanolic extract; AVOE: *Viburnum opulus* L. aqueous extract; MVOE: *Viburnum opulus* L. metanolic extract. ^1^ Values obtained in each measurement have been ranked in descending order. ^2^ Lower value indicates better overall performance.

## Data Availability

The original contributions presented in this study are included in the article/Supplementary Material. Further inquiries can be directed to the corresponding author.

## Supplementary Materials

The following supporting information can be downloaded at https://www.mdpi.com/article/10.3390/molecules30244677/s1: Figure S1: MS/MS spectra and chromatogram of malic acid in MVOE; Figure S2: MS/MS spectra and chromatogram of quinic acid in MVOE; Figure S3: MS/MS spectra and chromatogram of chlorogenic acid in MVOE; Figure S4: MS/MS spectra and chromatogram of chlorogenic acid dimer in AVOE; Figure S5: MS/MS spectra and chromatogram of procyanidin B2 in AVOE; Figure S6: MS/MS spectra and chromatogram of tricin in AVOE; Figure S7: MS/MS spectra and chromatogram of catechin in AVOE.

## Abbreviations

The following abbreviations are used in this manuscript:

VO	*Viburnum opulus* L.
VOFE	*Viburnum opulus* L. Fruit Extract
AVOE	Aqueous *Viburnum opulus* L. Extract
MVOE	Methanolic *Viburnum opulus* L. Extract
WA1–3 and 6	Waffles with 1–3-6g Aqueous *Viburnum opulus* L. Extract
WM1–3 and 6	Waffles with 1–3-6g Methanolic *Viburnum opulus* L. Extract
st	Standard
WAC	Water Absorption Capacity
OAC	Oil Absorption Capacity
CF	Cutting Force
PS	Puncture Strength
WS	Work of Shear
L*:	Lightness (0 = black, 100 = white)
a*	Green (–) to Red (+) axis
b*	Blue (–) to Yellow (+) axis
CW1	Control Waffles 1
CW2	Control Waffles 2
E	Ethanolic Extract
PBS	PBS Extract
GID	In Vitro Gastrointestinal Digestion Extract
TPC	Total Phenolic Content
TFC	Total Flavonoid Content
PAC	Phenolic Acid Content
GAE	Gallic Acid Equivalents
DW	Dry Weight
QE	Quercetin Equivalents
CAE	Caffeic Acid Equivalents
ABTS	2,2′-azino-bis(3-ethylbenzothiazoline-6-sulfonic acid) Radical Cation Decolorization Assay
DPPH	2,2-diphenyl-1-picrylhydrazyl Radical Scavenging Assay
OH Radical	Hydroxyl Radical Scavenging Activity
FRAP	Ferric Reducing Antioxidant Power
CHP	İron Chelation Power
TE	Trolox Equivalents
LOX	Lipoxygenase Inhibitory Activity
XO	Xanthine Oxidase Inhibitory Activity
