# Supplementary material for: Impact of Viburnum opulus L. Fruit Extracts on the Physicochemical, Sensory, and Bioactive Properties of Wheat Waffles
Source: Molecules. 2025 Dec 5;30(24):4677. doi: 10.3390/molecules30244677 (PMC12736300; doi:10.3390/molecules30244677)
Supplement: Supplementary file 1 [file molecules-30-04677-s001.zip › molecules-3987812-supplementary.pdf]

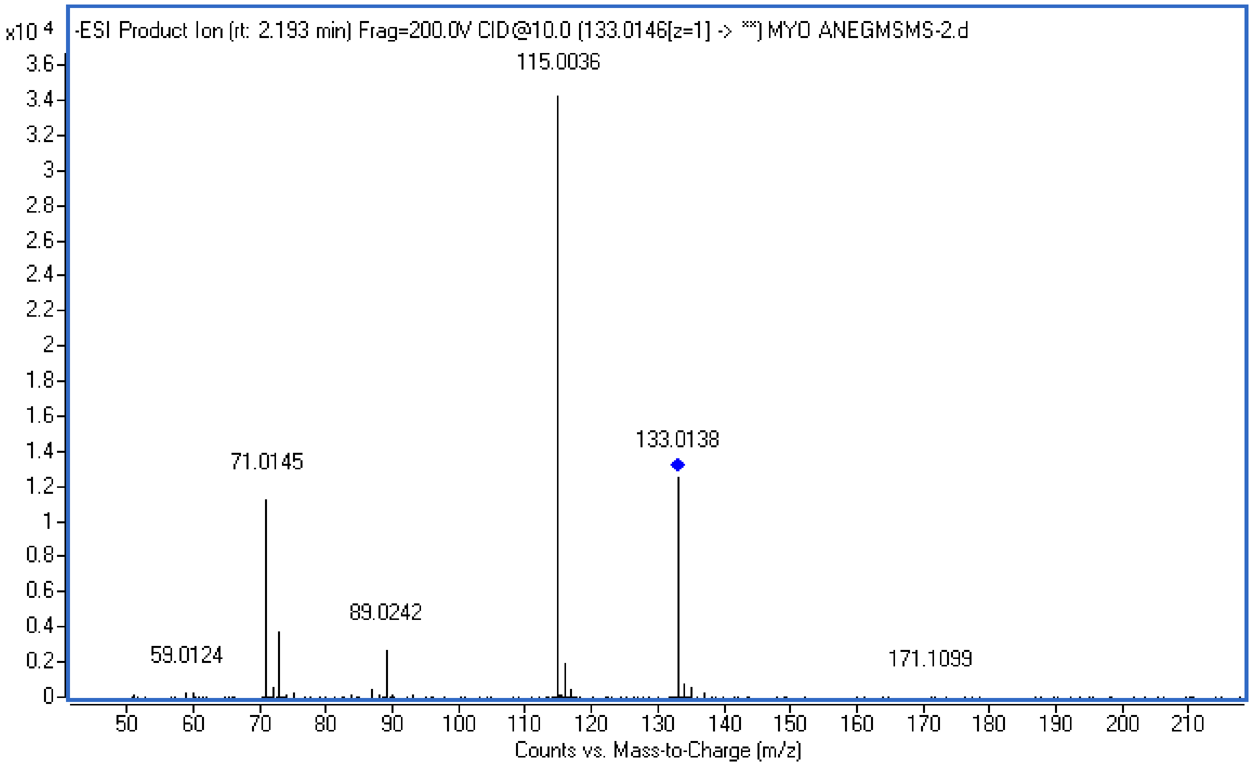

Figure S1. MSMS spectra and chromatogram of malic acid in MVOE.

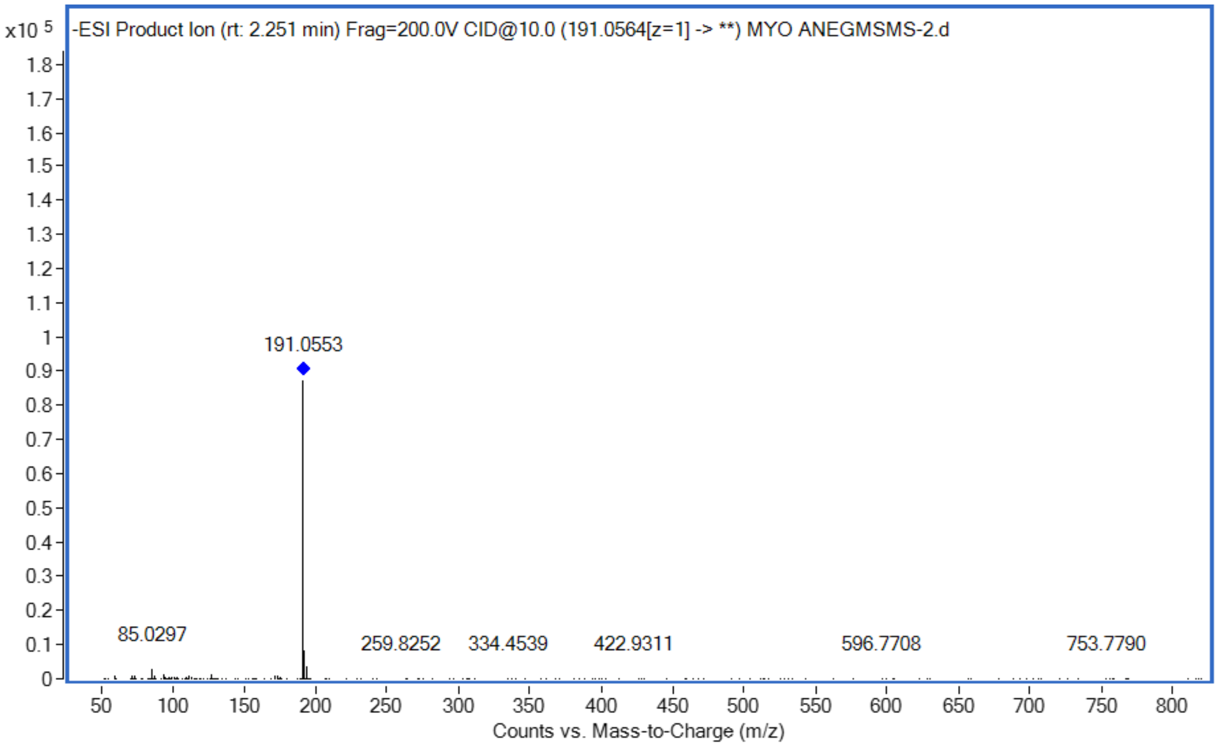

Figure S2. MSMS spectra and chromatogram of quinic acid in MVOE.

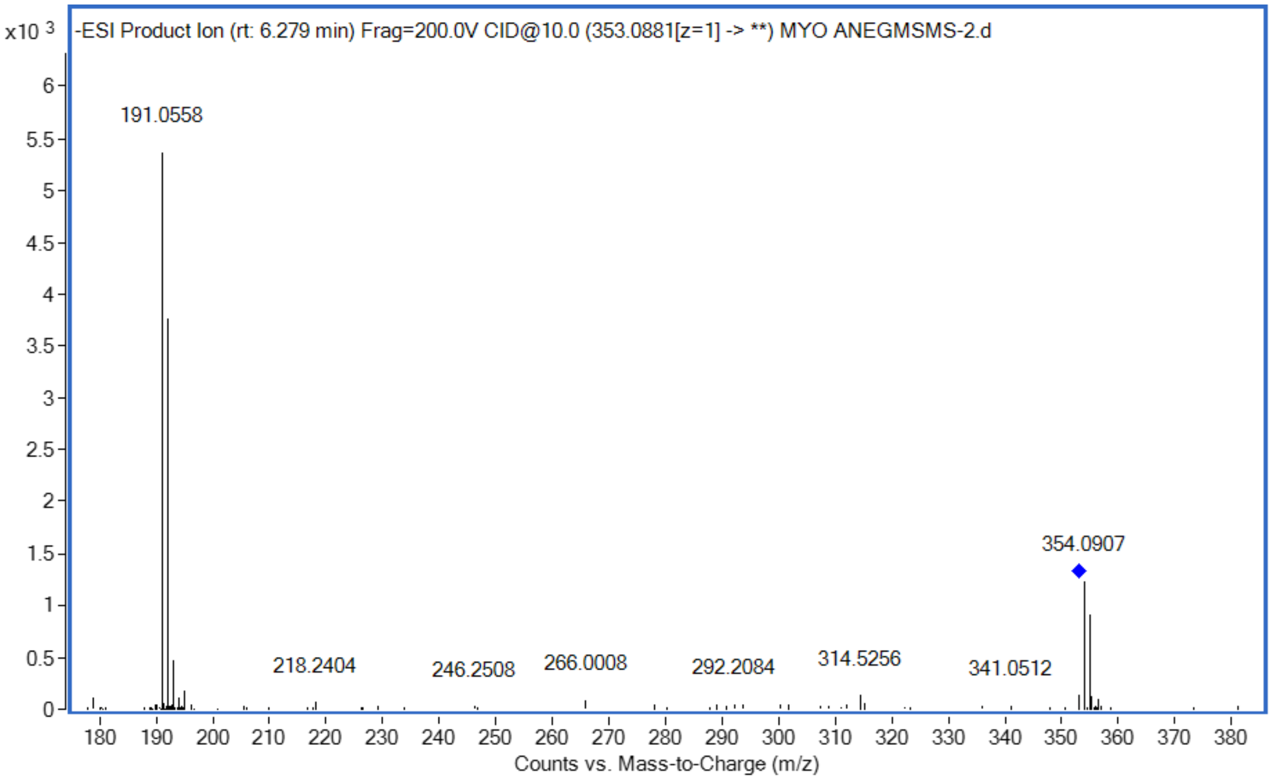

Figure S3. MSMS spectra and chromatogram of chlorogenic acid in MVOE.

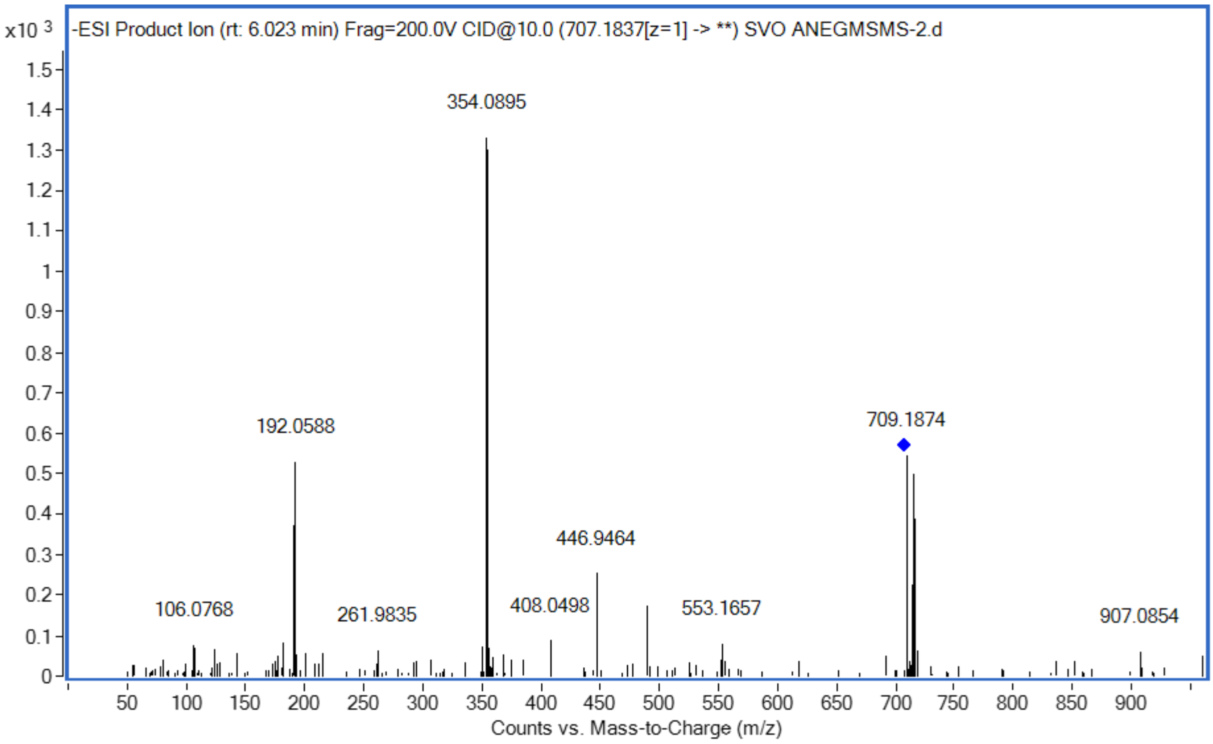

Figure S4. MSMS spectra and chromatogram of chlorogenic acid dimer in AVOE.

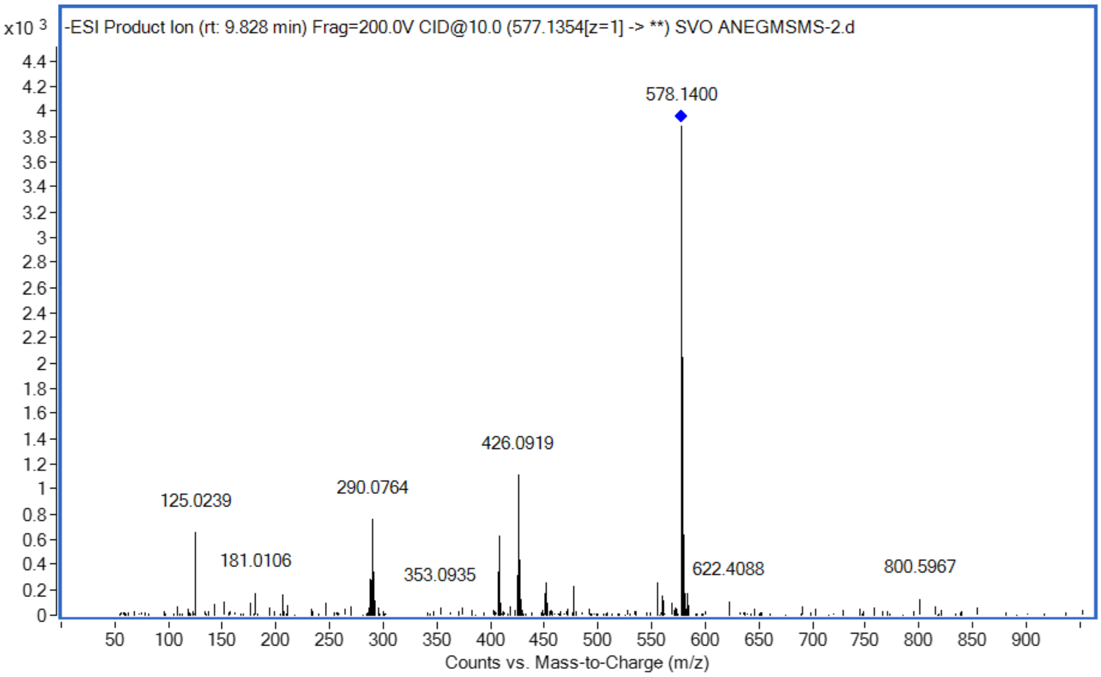

Figure S5. MSMS spectra and chromatogram of procyanidin B2 in AVOE.

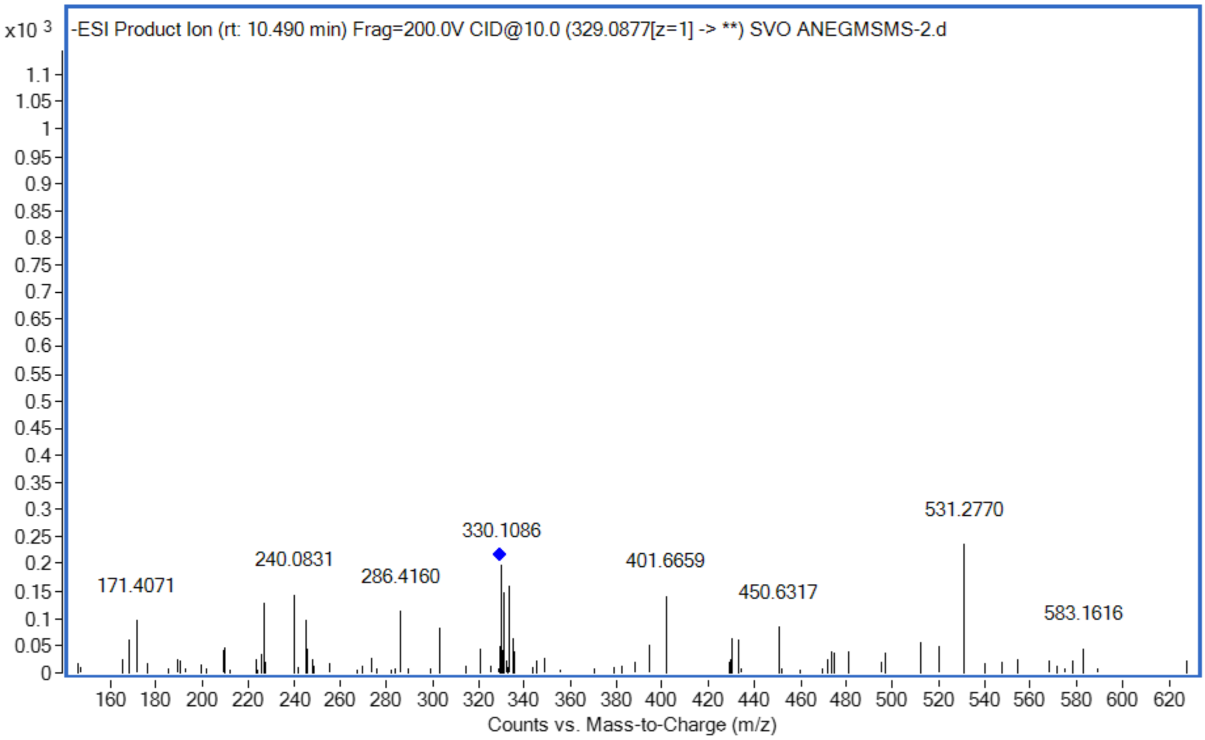

Figure S6. MSMS spectra and chromatogram of trisyn in AVOE.

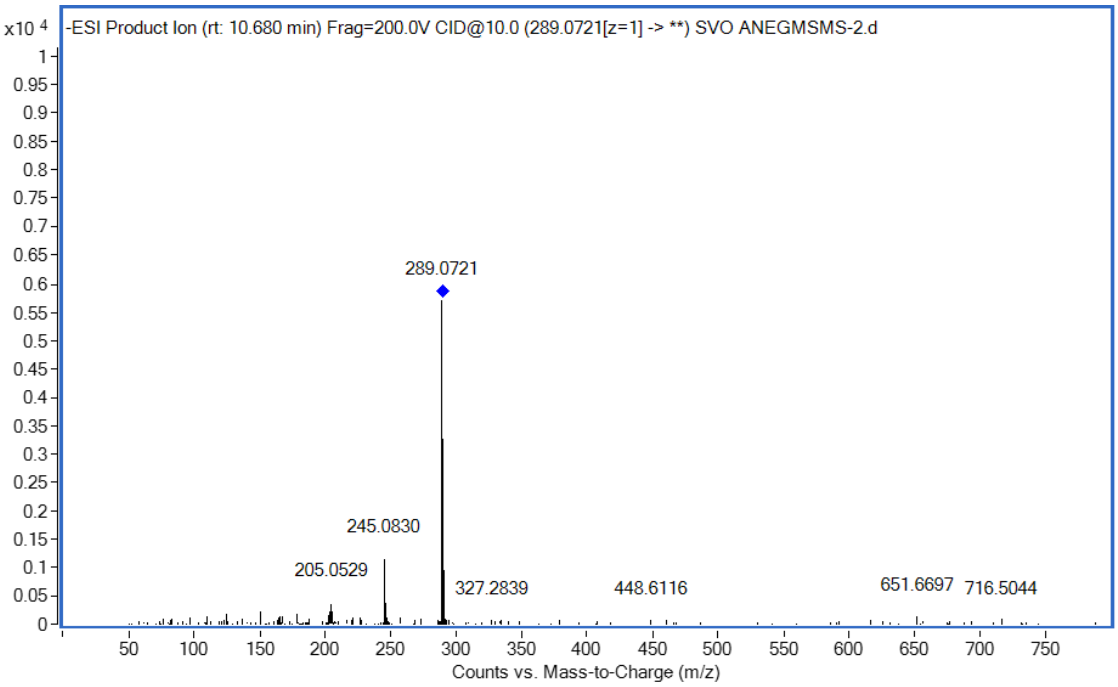

Figure S7. MSMS spectra and chromatogram of catechin in AVOE.
